# Effect of Dual-Task Motor-Cognitive Training in Preventing Falls in Vulnerable Elderly Cerebrovascular Patients: A Pilot Study

**DOI:** 10.3390/brainsci12020168

**Published:** 2022-01-27

**Authors:** Barbara Spanò, Maria G. Lombardi, Massimo De Tollis, Maria A. Szczepanska, Claudia Ricci, Alice Manzo, Simone Giuli, Lorenzo Polidori, Ivo A. Griffini, Fulvia Adriano, Carlo Caltagirone, Roberta Annicchiarico

**Affiliations:** Technology and Training Methods for Disability Care Laboratory, Department of Clinical and Behavioral Neurology, Santa Lucia Foundation IRCCS, 00179 Rome, Italy; lombardimariagiovanna@gmail.com (M.G.L.); m.detollis@hsantalucia.it (M.D.T.); szcmariaanna@gmail.com (M.A.S.); c.ricci@hsantalucia.it (C.R.); a.manzo@hsantalucia.it (A.M.); dott.simonegiuli@gmail.com (S.G.); l.polidori@hsantalucia.it (L.P.); i.griffini@hsantalucia.it (I.A.G.); fulvia.adriano@gmail.com (F.A.); c.caltagirone@hsantalucia.it (C.C.); r.annicchiarico@hsantalucia.it (R.A.)

**Keywords:** fall, older adult, cerebrovascular, dual-task, motor, cognitive, gait, balance, fear of falling, walking speed

## Abstract

Falling is a frequent and major clinical problem among older adults, as well as in patients with chronic cerebrovascular diseases (CVD). At present, sequential (mixed) and simultaneously (dual-task) motor-cognitive trainings are the best approaches to affording patients more autonomy in their everyday motor independence while reducing fall risks and consequences. The objective of this study was to evaluate the efficacy of an advanced and innovative dual-task motor-cognitive rehabilitation program on fall risks in vulnerable older persons with chronic CVD. To this purpose, 26 consecutive older fallers with chronic CVD were recruited, and completed a mixed motor-cognitive or a dual-task motor-cognitive training program. Each patient also underwent two test evaluations to assess balance, gait, fear of falling, and walking performance at pre-and post-intervention. We found that our experimental motor-cognitive dual-task rehabilitation program could be an effective method to improve walking balance, gait, walking speed, and fear of falling, while reducing the risk of falls in older people with chronic CVD. Furthermore, results show that the simultaneous motor-cognitive training is more effective than the sequential motor-cognitive training. Therefore, our study brings innovative data, which can contribute positively to the management of this population.

## 1. Introduction

Falling is a frequent and major clinical problem with potentially severe consequences in the elderly [1]. It is estimated that 20 to 30% of older adults fall each year [2]. Though not all falls are serious enough to require medical attention, it is known that all falls are predictors of future falls; they can lead to fear of falling, and can restrict a person’s activities of daily living [3,4].

In geriatric medicine, falls are considered a syndrome with multiple causes and contributors [5]. Chronic cerebrovascular diseases (CVD) are common diseases that endanger the health of older adults who are at high risk of imbalance or falls [6]. The overlap between CVD and falls is well established [4]. Both have a negative prognosis in elderly individuals in terms of mortality and morbidity [7]. Falls in patients with CVD are usually attributed to a combination of factors that may or may not be related to CVD, and CVD is just one of the many significant comorbidities that affect older adults [8]. Most older patients with CVD fall inside their homes; in fact, it has been reported that walking and transfers are the most frequent activities at the time of a fall [9]. A significant number of these falls result in soft tissue injuries, fractures, and the need for medical interventions. Falls can also lead to limitations in the performance of daily living activities, increased dependence, development of fear of falling, and low fall self-efficacy [10,11,12,13]. Thus, both serious and non-serious falls are still among the most common complications in CVD patients, and their increasing incidence poses a challenge for rehabilitation [4].

Few studies have examined fall prevention in CVD patients. However, interventions recommended for the general older population who have experienced falls have been reported [4]. Evidence highlights the efficacy of different types of interventions to prevent falls in the elderly [14,15]. It has been emphasized that programs to prevent falling should include multifactorial and exercise interventions, and should aim at improving cognitive functions [15,16].

The relationship between motor and cognitive skills and falls becomes evident if we consider that, in everyday life, walking is a very attention-demanding task that requires a high level of mobility skill and cognitive flexibility to plan and execute movements [17]. In this perspective, in dual-task situations, i.e., when an individual is required to walk and carry out another task simultaneously, such as during the activities of daily living, the increased cognitive demand may lead to worse performance on both tasks, and a greater risk of falling [18]. This problem is most prominent in older adults and in CVD patients because of their impaired motor and cognitive abilities [19,20,21].

Evidence suggests that an intervention which integrates cognitive training with standard motor training is the best approach to ensure that patients are more autonomous in their everyday motor activities, thus reducing the risk of falling and its consequences [6,16,22,23].

Furthermore, in a growing body of research, it has been shown that motor-cognitive dual-task training (i.e., cognitive rehabilitation training at the same time as exercise rehabilitation therapy) can enhance the effectiveness of patients’ physical motor function rehabilitation [6,24]. Additionally, there is increasing interest in the use of rehabilitation technology, such as virtual reality and robotics, to address balance and gait, to improve mobility, and prevent the risk of falling [23,24].

Thus, the aim of this study was to evaluate the efficacy of an advanced and innovative dual-task motor-cognitive rehabilitation program on the risk of falling in older persons with chronic CVD. This intervention model might have several advantages compared to standard rehabilitation protocols: the experience is engaging and captivating; the use of technologies allows greater flexibility in developing and proposing exercises adapted to the clinical aims, and with increasing levels of difficulty; the implementation of the exercises can be adapted to different types of cognitive profiles, so patients can experience a non-monotonous and interesting rehabilitation experience.

We hypothesized that when compared to separate traditional treatments (i.e., mixed motor and cognitive rehabilitation programs), our dual-task rehabilitation program involving cognitive and motor tasks simultaneously leads to a reduced risk of falling due to the improvement of balance and gait.

## 2. Materials and Methods

### 2.1. Participants and Study Design

Twenty-six consecutive older fallers with chronic CDV (i.e., ischemic and/or hemorrhagic stroke) presenting at the IRCCS Santa Lucia Foundation were randomly assigned to the mixed motor and cognitive (MixT), group (*n* = 13), or dual-task motor-cognitive training (DTT) group (*n* = 13).

All patients underwent a clinical screening that included the collection of medical history, history of previous falls, and the administration of the Tinetti Performance Oriented Mobility Assessment (POMA) [25,26].

Eligible participants were older adults (aged ≥ 65 years) with CVD and with a formal education of at least 5 years who met the inclusion eligibility criteria for risk of fall according to previous studies [16] (total POMA score ≤ 20 and/or at least one fall in the previous year).

The presence of major cognitive disturbances; a history of behavioral, psychiatric, and/or systemic disturbances; and/or receiving any rehabilitative treatment represented exclusion criteria.

### 2.2. Mixed Motor and Cognitive Training (MixT)

Those participants randomized into MixT underwent individual combined/mixed motor and cognitive treatments, resulting in 30 min of motor training and 30 min of cognitive training per session. The intervention program was administered through five weeks with three weekly sessions, totaling fifteen sessions. The MixT program is summarized in Table 1. Motor training consisted of a set of warm-up procedures (i.e., stretching and squatting), followed by exercises dedicated for the halftime of each session to balance and halftime to gait. Balance training consisted of exercises lifting up heels or tiptoes, lateral/forward shifting, and holding and flexion/extension exercises. Gait exercises involved forward and backward walking over the ground with several variants and without use of assistive devices. To prevent errors, verbal instructions were provided by the therapist to assist patients in exercise executing (e.g., “correct head position”, “correct feet position”, “correct balance”). All exercises were augmented in difficulty by increasing speed, repetition, and changing holding position (see Table A1 for a description of each motor exercise)

Cognitive training consisted of a set of exercises mainly focused on executive functions and attention (2/3 of the time of each training session). These were provided by trained cognitive therapists in an individual setting administered through a computerized touch-screen platform (either in a table or in an all-in-one desktop computer) [16]. Each exercise provided three increasing levels of difficulty (i.e., easy, intermediate, hard), adjusted by the therapists according to the subject’s capability, and starting with the easiest one and increasing after two sessions at the highest level. In particular, executive function exercises consisted of abstraction tasks, planning, and working memory (e.g., ordering at a restaurant following some rules; solving tasks on similarities, differences and analogies; sorting pictures guessing a covered criterion), whereas attention exercises consisted of selective and sustained attention tasks (e.g., paying attention to a target item among distractors). Exercises of other main cognitive functions (i.e., declarative memory, spatial orientation, constructional praxis, language, and abstract reasoning) were executed during the remaining 1/3 of the time of each training session [16]. (See Table A2 for a description of each cognitive exercise).

Intensity of each MixT session was patient-specific, with rest breaks provided upon therapist discretion and patients’ tolerance to activity.

### 2.3. Motor-Cognitive Dual-Task Training (DTT)

Those participants randomized into DTT underwent an individual experimental dual-task rehabilitation program. This consists of simultaneous administration of motor and cognitive tasks, stimulating patients to enhance both abilities, in a dedicated room, resulting in 40-min of training per session. All the exercises proposed were developed by the research team on the basis of specific technological properties of the applied aid.

The intervention program was administered through five weeks with three weekly sessions, totaling fifteen sessions. The DTT program is summarized in Table 1.

The first part of the DTT protocol (1/3 of the time of each training session) concerned the use of the sensory carpet (2 m), which presents diverse surfaces (medium density smooth, sandy, and cobbled), and a video projector. The exercises have been developed to stimulate balance and gait motor skills, and cognitive abilities, such as attention (alert, target visuospatial search), visual-spatial associative memory, language (reading and calculating), and executive functions (verbal judgments and cognitive estimates).

Examples of exercises are: following the traffic lights, the environmental scenarios inclusive of the congruent and incongruent sounds, the association of sounds and images to remember, walking while looking for numbers and making calculations.

**Figure 1:**
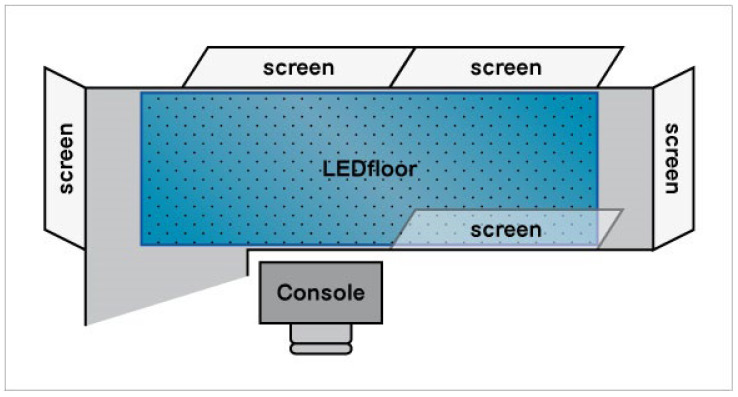
Dual-task room: five screens; one walkable led floor 4.5 m × 1.5 m; one audio/video controller console.

The second part of the protocol (2/3 of the time of each training session) concerned the use of a led floor (4.5 m × 1.5 m) and five video projectors (see Figure 1). We used an adapted version of the Walking Stroop carpet (WSC) used by Perrochon et al. [27] to detect cognitive impairment. The surface on which the patient walked was stable, and proposed different paths (follow the line, avoid the holes, center the box). The cognitive stimuli changed and required different cognitive functions: attention (visual–spatial research and attentional shifting), executive functions (interference inhibition, go/no go, updating), long- and short-term visuospatial memory, and language (denomination, calculation) (see Table A3 for a description of each cognitive exercise).

All the abilities are trained according to increasing levels of difficulty (except for the “Walking Stroop” and “Walking Trail Making Test” exercises, which do not present increasing levels of difficulty, but two phases that constitute the entire exercise to be administered consecutively). This allowed the personalization of the program according to patients’ needs.

To prevent errors, verbal instructions were provided by the therapist to assist patients in exercise executing (e.g., “correct head position”, “correct feet position”, “correct balance”).

Intensity of each DTT session was patient-specific, with rest breaks provided upon therapist discretion and patients’ tolerance to activity.

**Table 1:** Summarized description of MixT and DTT interventions.

	MixT	DTT
Training	Motor training (1) + cognitive training (2)	Motor-cognitive dual-task training (1)
Materials	(1) an empty room (2) a computerized touch-screen platform	(1) a dual-task room: three sensory carpets (2 m) (medium density smooth, sandy, and cobbled) and a video projector + five screens, a walkable led floor (4.5 m × 1.5 m), and an audio/video controller console.
Exercises	(1) Motor training: warm-up (stretching, squat with spread legs, squat with spread legs in anteroposterior) + functional balance (lift up heels, lift up tiptoes, lift up heels/tiptoes, lateral load shift, lateral load shift with contralateral leg flexion, lateral load shift with contralateral leg flexion and torso rotation, forward load shift, hip lift up opposite the support leg, load holding for 10 s, load holding with heel lift up, leg flexion/alternate leg flexion, leg flexion and extension/alternate leg flexion and extension, leg flexion and extension backwards, foot sliding forth and back) and gait (walking forward, walking forward oblique, walking forward flexing torso, walking forward oblique flexing torso, walking forward on a wide curve, walking backward, walking backward in line) (2) Cognitive training: executive functions and attention (similarities, differences, analogies, picture sort, be a piano player, take away menu, train guidance, guess who, n-black, remember the sequence) + memory (remember the picture, remember the melody, hide and find, remember the order, remember the design, find the pairs, who belongs where), constructional praxis (puzzle, copy of figures), language (synonymous, antonymous), logical reasoning (incomplete grids, symbol addiction, domino), and orientation (my home, travelling in Europe) exercises.	(1) Motor/cognitive training: with sensory carpets (subtraction, sounds, letters and words, go/no go), and with walkable led floor (Walking Stroop, Walking Trail Making Test, avoid the holes, shopping list) exercises.
How much	15 sessions: (1) motor training + (2) cognitive training, 60 (30 + 30) min/day, 3 days/weeks, 5 weeks.	15 sessions: (1) simultaneous motor/cognitive tasks, 40 min/day, 3 days/weeks, 5 weeks.
Who provided	Both MixT and DTT interventions were carried out by rehabilitation therapists with over 3 years of experience. Verbal assistance was provided by the intervention therapist as needed (e.g., to correct performance of exercises). Intervention measures were developed by experimental researchers.	
How	Both MixT and DTT interventions was conducted in one-on-one training sessions daily.	
Tailoring	The intervention of both MixT and DTT groups was adjusted according to the subject’s capability. Before training, therapists make a simple and rapid assessment of patients to select the personalized intervention intensity suitable for patients. Programs can also be adjusted according to patients’ own preferences. The intensity of each session was patient-specific, with rest breaks provided upon therapist discretion and patients’ tolerance to activity. Each exercise provided increasing levels of difficulty adjusted by the therapists, consistent with the subject’s capability.	

Abbreviations: MixT: combined motor and cognitive training; DTT: motor-cognitive dual-task training. See text and Appendix A for more details.

### 2.4. Outcomes

To assess the impact of different training on risk of falls, each patient underwent two evaluations, one within 1 week before the start of the study (MixT or DTT), and one within 1 week after the intervention (5 weeks later). Assessments were performed through standardized scales, and included: (1) evaluation of motor performance aimed at balance and gait with Tinetti Performance Oriented Mobility Assessment (POMA) for balance (POMA-B) and gait (POMA-G) [25,26]; (2) evaluation of fear of falling with the Falls Efficacy Scale-International (FES-I) [10,28]; (3) evaluation of physical performance with the six-minute walking test (6-MWT) [29,30], and gait speed (calculated as 6-MWT distance in meters divided by 360 s).

### 2.5. Data Analysis

Statistical analyses were carried out using IBM SPSS, version 21.0 (SPSS Inc., Chicago, IL, USA).

Continuous variables are reported as mean ± standard deviation. Categorical variables are reported as frequency and percentage. Differences in the demographic data of the groups were tested using either the Mann–Whitney U test or χ^2^ test according to the level of measurement.

The within-group effects (i.e., the difference in the outcomes observed between T0 and T1) were examined by employing the Wilcoxon signed-ranks test. We adopted this nonparametric test because the sample size was small. We also report the Z-score to represent the within-group effect size.

Change values for the outcome measures were calculated by subtracting the baseline data from the post-intervention data. To analyze between-group improvement, the change values were analyzed using the Mann–Whitney U test.

For the outcome, after the Bonferroni correction, the statistically significant threshold was set at *p* < 0.008.

## 3. Results

All recruited patients (13 + 13) completed a training program (MixT or DTT), underwent two test evaluations (pre- and post- intervention), and were included in the statistical analysis. During the study, no adverse events, such as a fall, were encountered. Patients’ demographic and clinical results at baseline are shown in Table 2. MixT patients and DTT patients were well matched for age (U = 56.00, Z = −1.46, *p* = 0.15). There was a significant difference in sex distribution (χ^2^ = 5.57, df = 1, *p* = 0.02).

No significant differences between groups were found for any of the outcome measures at the pre-intervention assessment (POMA total score: U = 83.50, Z = −0.05, *p* = 0.96; POMA-B: U = 78.00, Z = −0.34, *p* = 0.76; POMA-G: U = 68.00, Z = −0.86, *p* = 0.42; FES-I: U = 75.00, Z = −0.49, *p* = 0.65; 6-MWT: U = 70.00, Z = −0.74, *p* = 0.48; gait speed: U = 70.00, Z = −0.74, *p* = 0.48).

**Figure 2:**
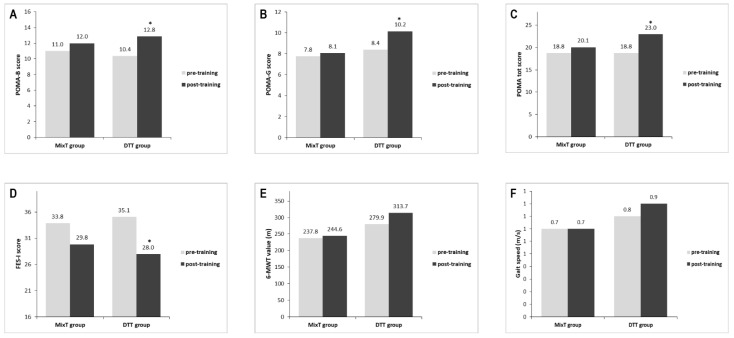
Outcome performance after different training protocols. (**A**) POMA-B, (**B**) POMA-G, (**C**) POMA total, (**D**) FES-I, (**E**) 6-MWT, (**F**) Gait speed. * Significant intra-group difference *p* < 0.01. Abbreviations: MixT: combined motor and cognitive training; DTT: motor-cognitive dual-task training; POMA tot: Tinetti Performance Oriented Mobility Assessment total score; POMA-B: POMA balance; POMA-G: POMA gait; FES-I: Falls Efficacy Scale-International; 6-MWT (m): 6-min walk test (meters).

Figure 2 shows outcome performances after different training protocols. In the DTT group, a significant improvement was found in POMA total score (Z = −3.20, *p* = 0.001), POMA-B (Z = −3.06, *p* = 0.002), POMA-G (Z = −3.13, *p* = 0.002), and FES-I (Z = −3.07, *p* = 0.002), but not in 6-MWT (Z = −1.40, *p* = 0.16) performance or in gait speed (Z = −1.49, *p* = 0.14), compared to pre-training. In the MixT group, no significant improvements from pre-training measurements were observed (POMA total score: Z = −2.03, *p* = 0.04; POMA-B: Z = −2.01, *p* = 0.04; POMA-G: Z = −1.41, *p* = 0.16; FES-I: Z = −1.64, *p* = 0.10; 6-MWT: Z = −1.06, *p* = 0.29; gait speed: Z = −1.16, *p* = 0.25).

Table 3 shows the measured outcomes while performing serial subtraction at pre- and post-intervention for both training groups. When comparing DTT and MixT groups, a post-training significant improvement was observed in DTT group with respect to the POMA total score (U = 21.50, Z = −3.31, *p* = 0.001) and POMA-G (U = 23.50, Z = −3.28, *p* = 0.001). Conversely, no difference was found when comparing POMA-B (U = 43.00, Z = −2.17, *p* = 0.03), FES-I (U = 50.50, Z = −1.76, *p* = 0.08), 6-MWT (U = 77.00, Z = −3.39, *p* = 0.72), or gait speed (U = 77.00, Z = −3.39, *p* = 0.72) post-training change values.

Moreover, after intervention, 6-MWT and gait speed were generally maintained in the MixT group, whereas improvements of 33.8 m and 0.10 m/s, respectively, were seen in the DTT group (see Table 3).

## 4. Discussion

In this study, we aimed to evaluate the efficacy of our experimental, motor-cognitive, dual-task rehabilitation program (DTT), developed within a dual-task room, on the risk of falling in vulnerable older patients with chronic CVD, compared to a conventional mixed motor-cognitive training (MixT).

It is known that both MixT (i.e., motor and cognitive trainings are conducted separately) and DTT (i.e., motor and cognitive trainings are conducted simultaneously) are effective in improving physical performance (e.g., balance, gait, walking speed) and fear of falling in older adults and stroke patients [6,16,31,32,33,34]. However, clinical evidence which shows that DTT is more effective than its sequential counterpart is lacking.

Furthermore, to the best of our knowledge, this is the first pilot study that compares the effects of a simultaneous vs. a sequential motor-cognitive training program in reducing the risk of falling in older individuals with chronic CVD. Therefore, our study provides innovative data that can contribute positively to the management of this population.

According to our findings, the experimental motor-cognitive dual-task rehabilitation program is an effective method for improving walking balance, gait, and walking speed, as well as reducing the fear of falling. It also leads to a reduced risk of falls in older people with chronic CVD. Furthermore, DTT is a more effective approach than MixT.

Results show that both groups (i.e., the experimental DTT and the conventional MixT) improved, but only the dual-task intervention was effective in mobility (POMA-total) in both balance (POMA-B) and gait (POMA-G) components, and in fear of falling (FES-I).

However, the superiority of this treatment over the conventional one was specifically found only for mobility (POMA-tot) and its gait component (POMA-G).

This slight superiority obtained in the DTT group in the studied variables seems to be related to the specificity of this training, because it incorporates dual-task cognitive-motor aspects that are so important for this population.

First, in the case of the POMA test, similar findings on the balance component (POMA-B) only suggest that our DTT intervention is more effective in enhancing dynamic/walking balance than static/standing balance. In this regard, an improvement in the general balance condition may have been promoted by weight transfer and dynamic postural adaptation. Although we can only speculate about these findings, we propose that the POMA-B is more sensitive for assessing the static balance component. In fact, in the POMA-B, balance is assessed while the person being evaluated is sitting, arising, standing (immediate and prolonged), and turning. On the other hand, the POMA-G component test, which includes initiation, step length and height, step symmetry, step continuity, walking path, trunk sway, and walking stance (width), could be an indirect measure of dynamic/walking balance.

Consistent with this speculation, it was reported that an unstable support surface, such as that used in our DTT protocol, stimulates tactile sensation, vestibular sensations, proprioceptive sensations, etc., to induce posture and balance reactions, and, thus, promote dynamic stability [35].

Furthermore, a recent RCT-based meta-analysis [6] reported that the improvement effect of cognitive-motor dual-task training on the walking balance of chronic stroke patients was significantly better than that in the control group.

Second, although the DTT intervention did not statistically improve walking performance (6-MWT and/or gait speed), it is noteworthy that, in the DTT group, walking endurance (6-MWT distance) and gait speed were improved by 34 m and 0.10 m/s, respectively. Similar to our findings, Barboza et al., [36] and Liu et al. [32] found no significant differences between the group that received the motor plus cognitive training [36] or the cognitive-motor dual-task [32] than the group that received only motor training and/or the motor-motor dual-task. Nevertheless, our findings on walking performance are interesting, considering that it has been reported that an improvement of 0.10 m/s for gait speed, and an improvement of 20 m for 6MWD, are considered clinically meaningful changes [37].

Third, regarding the FES-I results, only the DTT intervention was effective in reducing fear of falling in older people with chronic CVD. It is also relevant that the patients who underwent the DTT obtained an FES-I score of 28, which is considered the cut-off score for classifying fallers and non-fallers [10]. On the other hand, these findings fit with our other study results, and are in line with a parallel outcome in the literature. In a recent study, Sampaz Mujdeci et al. [3] showed that fear of falling negatively affects balance performance; Lopes et al. [38] found a correlation between fear of falling and dynamic balance and mobility; and another study [39] found a very high relationship among fear of falling, functional mobility, and balance. Likewise, here, we found that changes in fear of falling parallel changes in balance and mobility performance.

This study also suffers from a series of limitations. First, the sample size is small. This limits confidence in the effects that were observed, and makes it difficult to generalize the results. A larger, randomized, controlled clinical trial is needed to validate the reported benefits of the dual-task training protocols reported in the current study. Second, because there was no follow-up test, it was impossible to demonstrate whether improvements could be maintained. Third, the lack of a cognitive assessment did not allow us to evaluate the effect of treatment on cognitive performance. Indeed, future research should consider this issue.

## 5. Conclusions

This study presents an alternative intervention for older people with CVD. The preliminary results suggest that our DTT can have (has) an adequate influence on the improvement of walking balance, gait, walking endurance and speed, and fear of falling. Future studies should replicate this pilot study by employing a larger sample, so that its effects can be more confidently evaluated.

## Figures and Tables

**Table 2:** Patients’ demographic and clinical results at baseline.

	MixT Group (*n* = 13)	DTT Group (*n* = 13)	*p* Value
Age (years) ^a^	79.8 ± 8.7	75.4 ± 5.5	0.15
Sex (male/female) ^b^	3/10	9/4	0.02
POMA tot ^a^	18.8 ± 6.6	18.8 ± 2.6	0.96
POMA-B ^a^	11.0 ± 4.6	10.4 ± 2.0	0.76
POMA-G ^a^	7.8 ± 2.6	8.4 ± 1.7	0.42
FES-I ^a^	33.8 ± 12.4	35.1 ± 11.4	0.65
6-MWT (m) ^a^	237.8 ± 195.0	279.9 ± 139.5	0.48
Gait speed (m/s) ^a^	0.7 ± 0.5	0.8 ± 0.4	0.48

Abbreviations: MixT: combined motor and cognitive training; DTT: motor-cognitive dual-task training; *p* value, between-group difference; POMA tot: Tinetti Performance Oriented Mobility Assessment total score; POMA-B: POMA balance score; POMA-G: POMA gait score; FES-I: Falls Efficacy Scale-International score; 6-MWT: 6-min walk test; m = meters; s = seconds. ^a^ Values are mean ± SD, ^b^ Values are number. See text for more details.

**Table 3:** Outcome performance after different training protocols.

	MixT Group (*n* = 13)		DTT Group (*n* = 13)		Inter-Group Differences
	Pre	Post	Pre	Post	
POMA-tot ^a^	18.8 ± 6.7	20.1 ± 6.4	18.8 ± 2.6	23.0 ± 2.6	
Change values ^b^		1.3 ± 1.9		4.2 ± 1.4	*p* = 0.001
POMA-B ^a^	11.0 ± 4.6	12.0 ± 4.1	10.4 ± 2.0	12.8 ± 2.0	
Change values ^b^		1.0 ± 1.7		2.5 ± 1.6	*p* = 0.03
POMA-G ^a^	7.8 ± 2.6	8.1 ± 2.9	8.4 ± 1.7	10.2 ± 1.2	
Change values ^b^		0.3 ± 0.8		1.8 ± 1.1	*p* = 0.001
FES-I ^a^	33.8 ± 12.4	29.8 ± 9.0	35.1 ± 11.4	28.0 ± 9.8	
Change values ^b^		−4.0 ± 9.1		−7.1 ± 6.3	*p* = 0.08
6-MWT (m) ^a^	237.8 ± 195.1	244.6 ± 205.4	279.9 ± 139.5	313.7 ± 106.0	
Change values ^b^		6.7 ± 44.4		33.8 ± 75.1	*p* = 0.72
Gait speed (m/s) ^a^	0.7 ± 0.5	0.7 ± 0.6	0.8 ± 0.4	0.9 ± 0.3	
Change values ^b^		0.0 ± −0.1		0.1 ± 0.2	*p* = 0.72

Abbreviations: MixT: combined motor and cognitive training; DTT: motor-cognitive dual-task training; *p* value, between-group difference; POMA-tot: Tinetti Performance Oriented Mobility Assessment total score; POMA-B: POMA balance score; POMA-G: POMA gait score; FES-I: Falls Efficacy Scale-International score; 6-MWT: 6-min walk test; m = meters; s = seconds. ^a^ Values are mean ± SD. ^b^ Change values were calculated by subtracting the pre-training data from the post-training data. See text for more details.

**Figure A1:**
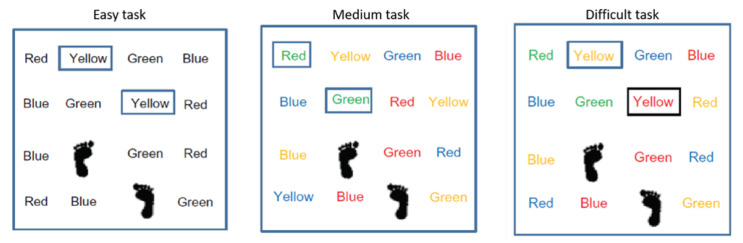
Example of a walkable led floor representation during an easy, medium, or difficult DTT Walking Stroop task. See Table A3 for more details. A figure was reproduced, with the permission of the authors, from Figure 1B–D in Clinical interventions in Aging, Walking Stroop carpet: an innovative dual-task concept for detecting cognitive impairment, Clinical Interventions in Aging 2013, 8, 317–328 by Perrochon et al. [27].

## Appendix A

**Table A1:** Exercises of motor training in the MixT protocol.

Warm-up:
Stretching
Squat with spread legs
Squat with spread legs in anteroposterior
Balance:
Lift up heels
Lift up tiptoes
Lift up heels/tiptoes
Lateral load shift
Lateral load shift with contralateral leg flexion
Lateral load shift with contralateral leg flexion and torso rotation
Forward load shift
Hip lift up opposite the support leg
Load holding for 10 s
Load holding with heel lift up
Leg flexion/alternate leg flexion
Leg flexion and extension/alternate leg flexion and extension
Leg flexion and extension backwards
Foot sliding forth and back
Gait:
Walking forward
Walking forward oblique
Walking forward flexing torso
Walking forward oblique flexing torso
Walking forward on a wide curve
Walking backward
Walking backward in line

Abbreviations: MixT: combined motor and cognitive training.

**Table A2:** Exercises of cognitive training in the MixT protocol.

Executive functions and attention:
Similarities: the user is asked to select the correct sentence among three describing the similarity between two concepts.
Differences: the user is presented with a couple of words/pictures; he has to explain in what way they are different, selecting one of three sentences.
Analogies: the user is given one pair of related words/pictures, and another word/picture without its pair. The user must choose a word/picture that has the same relationship to the word/picture as the first pair.
Picture sort: there are two boxes of different color. The images are presented one by one on top of the screen, and the user has to drag and drop each image to the correct box by discovering the hidden rule inferred by the feedback given after each choice.
Be a piano player: the user is presented with a piano playing some notes. When a note plays, the corresponding key lights up, and the user is required to play the same note, pressing the corresponding key. Notes are presented at increasing speed.
Take away menu: the user must order from a take-away menu following some rules (e.g., maximum price, foods not to be ordered).
Train guidance: the user is presented a group of persons on the screen moving in different directions. The aim of the task consists of choosing the direction of the central person, suppressing automatic response to the stimuli.
Guess who: the user is asked to eliminate candidates, and correctly guess the mystery person, chosen using the cues provided.
N-back: a sequence of pictures is presented. The user is asked to touch the screen when a picture matches with a picture presented n steps earlier in the sequence (e.g., 1, 2, or 3…). On the side, a green flag or a red X appear based on whether the answer is right or wrong.
Remember the sequence: the user is presented with an array with a start box colored. All possible pictures that can be displaced on the array are presented at the bottom of the array. The sequence starts with one picture positioned in one box next to the start box. Then, the clear array is re-presented, and the user has to drag the correct picture into the correct position. Then, two other pictures are presented, each time in a different sequence, and so on. The user is notified upon making a mistake, and there are no time limits to complete each sequence.
Memory
Remember the picture: the task is presented with a photo album of one large and several smaller photos. The largest photo—the one to remember—is replaced with another one after a few seconds. Among the smaller photos is the one that was shown previously, and needs to be chosen from. The user is notified when making a mistake, and then the game starts from the beginning.
Remember the melody: the user is presented with a piano playing some notes, with the corresponding key lighting up. The user is asked to remember in which specific sequence the notes were played, and to reproduce the melody. The user is notified upon making a mistake, and then the game starts from the beginning.
Hide and find: The user is shown of a fully-furnished and fully-decorated room, and is asked to hide 5–10 items in there. After a 15–20 min delay, the user is asked to recall where he has hidden the various objects.
Remember the order: the user is shown a menu, and is asked to memorize a list of dishes. Immediately after, the user is asked to recognize the studied materials among distracters.
Remember the design: the user is shown a design drawn on a 9-dot matrix, and is asked to encode it. Subsequently, the user is asked to reproduce the pattern on screen.
Find the pairs: the user is shown a grid of paired cards. After the cards are covered, the user is asked to find the matching pictures.
Who belongs where: the user has to choose the correct profession of famous people, dragging the pictures in the right category among two alternatives.
Constructional praxis
Puzzle: the user is shown pieces of a puzzle, and is asked to put them onto a grid in order to make the picture. There is no time limit to complete the game.
Copy of figures: the user is asked to copy the geometrical figures using fingers.
Language
Synonymous: a set of words are presented in two different lists. The user is required to draw a line with his finger between the words on the left and their synonyms on the right.
Antonymous: a set of words are presented in two different lists. The user is required to draw a line with his finger between the words on the left and their antonyms on the right.
Logical reasoning
Incomplete grids: the user is asked to complete the image representing a visuo-spatial pattern by inserting the right tile in a multiple choice.
Symbol addition: the user is asked to solve arithmetic operations using symbols instead of digits.
Domino: the user is asked to pair identical dominos by placing each tile next to the corresponding one.
Orientation
My home: the user has to find a way to move a person into a house following different indications to cover a specific trail.
Travelling in Europe: the user is asked to make a virtual tour selecting several countries following a specific sequence.

Abbreviations: MixT: combined motor and cognitive training.

**Table A3:** Exercises of motor-cognitive dual-task training in the DTT protocol.

Sensory carpets (medium density smooth, sandy, and cobbled)
Subtraction: the user is asked to walk forward on a carpet by subtracting from 50, one (i.e., 50-1; easy), two (i.e., 50-2; medium), or three (i.e., 50-3 difficult) numbers.
Sounds: the user is asked to walk forward on a carpet by listening a sound (e.g., sound of bell, phone, intercom, train, rain, etc.; easy), by listening a sound and naming it (medium), or by listening a sound and making a judgment (i.e., natural/artificial, coherent/incoherent) on it (difficult).
Letters and words: the user is asked to walk forward on a carpet by saying the letters of the alphabet out loud (a letter at every step; easy), by saying words that begin with a certain letter (medium) out loud, or by saying words that belong to a certain category out loud (e.g., animals; difficult).
Go/No go: the user is asked to walk forward on a carpet respecting congruent (i.e., walk at a green light, and stop at a red light; easy), or incongruent (i.e., stop at a green light, and walk at a red light; easy) signals projected on a screen.
Walking Stroop: the user is asked to walk forward a walkable led floor with black words naming colors (e.g., red, yellow, green, blue), following words naming a color (e.g., yellow), and saying the color out loud (e.g., yellow) (easy); or to walk forward on a walkable led floor with colored words, naming correspondent (e.g., word “green” colored in green) and non-correspondent colors (e.g., word “red” colored in green), following words’ colors in a certain color (e.g., all words colored in green), and saying the followed color out loud (e.g., green) (medium); or to walk forward on a walkable led floor with colored words, naming correspondent (e.g., word “yellow” colored in yellow) and non-correspondent colors (e.g., word “yellow” colored in red), following words naming a certain color (e.g., all words “yellow”) and saying the followed color out loud (e.g., “yellow”) (difficult). (Figure A1)
Walking Trail Making Test: the user is asked to walk forward on a walkable led floor with numbers or numbers + letters following in ascending order (from 1 to 20 and/or from a to z) numbers (easy), or numbers + letters (difficult).
Avoid the holes: the user is asked to walk forward on a walkable led floor that simulates a street, avoiding holes that appear on one side (easy) or on both sides (medium) of the street, and by following the directions on signs projected on a screen (e.g., stop, raise your arms).
Shopping list: the user is asked to walk forward on a walkable led floor that simulates a street by looking at (projected on a screen) or listening to some shopping list items (e.g., milk, eggs, soap). She/he is asked to cross the street by remembering the shopping list.

Abbreviations: DTT: motor-cognitive dual-task training.
