# Correction: Spanò et al. Effect of Dual-Task Motor-Cognitive Training in Preventing Falls in Vulnerable Elderly Cerebrovascular Patients: A Pilot Study. *Brain Sci.* 2022, *12*, 168

**DOI:** 10.3390/brainsci14040370

**Published:** 2024-04-11

**Authors:** Barbara Spanò, Maria G. Lombardi, Massimo De Tollis, Maria A. Szczepanska, Claudia Ricci, Alice Manzo, Simone Giuli, Lorenzo Polidori, Ivo A. Griffini, Fulvia Adriano, Carlo Caltagirone, Roberta Annicchiarico

**Affiliations:** Technology and Training Methods for Disability Care Laboratory, Department of Clinical and Behavioral Neurology, Santa Lucia Foundation IRCCS, 00179 Rome, Italy; lombardimariagiovanna@gmail.com (M.G.L.); m.detollis@hsantalucia.it (M.D.T.); szcmariaanna@gmail.com (M.A.S.); c.ricci@hsantalucia.it (C.R.); a.manzo@hsantalucia.it (A.M.); dott.simonegiuli@gmail.com (S.G.); l.polidori@hsantalucia.it (L.P.); i.griffini@hsantalucia.it (I.A.G.); fulvia.adriano@gmail.com (F.A.); c.caltagirone@hsantalucia.it (C.C.); r.annicchiarico@hsantalucia.it (R.A.)

## Missing Citation

In the original publication [1], Perrochon et al. [27] was not cited. The citation has now been inserted in *Section 2.3. Motor-Cognitive Dual-Task Training (DTT), Paragraph 5* and should read:

“We used an adapted version of the Walking Stroop carpet (WSC) used by Perrochon et al. [27] to detect cognitive impairment.”

The citation has now been inserted in Figure A1 legend and should read:

**Figure A1.** Example of a walkable led floor representation during an easy, medium, or difficult DTT Walking Stroop task. See Table A3 for more details. A figure was reproduced, with the permission of the authors, from Figure 1B–D in Clinical interventions in Aging, Walking Stroop carpet: an innovative dual-task concept for detecting cognitive impairment, Clinical Interventions in Aging 2013, 8, 317–328 by Perrochon et al. [27].

The newly added reference appears below:

27. Perrochon, A.; Kemoun, G.; Watelain, E.; Berthoz, A. Walking Stroop carpet: An innovative dual-task concept for detecting cognitive impairment. *Clin. Interv. Aging* **2013**, *8*, 317–328. https://doi.org/10.2147/CIA.S38667.

## Text Correction

There was a minor typographical error in the original publication [1]. In Section 2.3. Motor-Cognitive Dual-Task Training (DTT), Paragraph 5, “a led wall” should be “a led floor”, the correct sentence appears below:

“The second part of the protocol (2/3 of the time of each training session) concerned the use of a led floor (4.5 m × 1.5 m) and five video projectors (see Figure 1).”

With these corrections, the order of some references has been adjusted accordingly. The authors state that the scientific conclusions are unaffected. This correction was approved by the Academic Editor. The original publication has also been updated.
